# Exploiting MeSH indexing in MEDLINE to generate a data set for word sense disambiguation

**DOI:** 10.1186/1471-2105-12-223

**Published:** 2011-06-02

**Authors:** Antonio J Jimeno-Yepes, Bridget T McInnes, Alan R Aronson

**Affiliations:** 1 National Library of Medicine, 8600 Rockville Pike, Bethesda, MD 20894, USA; 2 Department of Pharmacology, University of Minnesota Twin Cities, Minneapolis, MN 55155, USA

## Abstract

**Background:**

Evaluation of Word Sense Disambiguation (WSD) methods in the biomedical domain is difficult because the available resources are either too small or too focused on specific types of entities (e.g. diseases or genes). We present a method that can be used to automatically develop a WSD test collection using the Unified Medical Language System (UMLS) Metathesaurus and the manual MeSH indexing of MEDLINE. We demonstrate the use of this method by developing such a data set, called MSH WSD.

**Methods:**

In our method, the Metathesaurus is first screened to identify ambiguous terms whose possible senses consist of two or more MeSH headings. We then use each ambiguous term and its corresponding MeSH heading to extract MEDLINE citations where the term and only one of the MeSH headings co-occur. The term found in the MEDLINE citation is automatically assigned the UMLS CUI linked to the MeSH heading. Each instance has been assigned a UMLS Concept Unique Identifier (CUI). We compare the characteristics of the MSH WSD data set to the previously existing NLM WSD data set.

**Results:**

The resulting MSH WSD data set consists of 106 ambiguous abbreviations, 88 ambiguous terms and 9 which are a combination of both, for a total of 203 ambiguous entities. For each ambiguous term/abbreviation, the data set contains a maximum of 100 instances per sense obtained from MEDLINE.

We evaluated the reliability of the MSH WSD data set using existing knowledge-based methods and compared their performance to that of the results previously obtained by these algorithms on the pre-existing data set, NLM WSD. We show that the knowledge-based methods achieve different results but keep their relative performance except for the Journal Descriptor Indexing (JDI) method, whose performance is below the other methods.

**Conclusions:**

The MSH WSD data set allows the evaluation of WSD algorithms in the biomedical domain. Compared to previously existing data sets, MSH WSD contains a larger number of biomedical terms/abbreviations and covers the largest set of UMLS Semantic Types. Furthermore, the MSH WSD data set has been generated automatically reusing already existing annotations and, therefore, can be regenerated from subsequent UMLS versions.

## Background

*Word Sense Disambiguation *(WSD) is the task of automatically identifying the appropriate sense (or concept) of an ambiguous word; for example, the term *cold *could refer to the temperature or a virus depending on the context in which it is used. Not being able to identify the intended concept of an ambiguous word negatively impacts the accuracy of biomedical applications such as medical coding and indexing which are becoming essential in the biomedical world due to the growing amount of information that is available to researchers.

Evaluation and comparison of WSD methods in the biomedical domain is difficult because many freely available test collections only cover a specific type of entity. For example, a segment of the BioCreative [1] data set contains mappings of genes names from text to their appropriate gene identifier, the E. Coli corpus [2] contains mappings of E. Coli mentions to the Universal Protein Resource (UniProt), the Arizona Disease Corpus (AZDC) [3,4] contains mappings of disease entities to concepts in the UMLS, and Guadan, et al. [5] map abbreviations found in biomedical abstracts to their proper expansion.

Most test collections contain only a limited number of ambiguous terms and corresponding instances containing the resolved mapping of the term. For example, the National Library of Medicine's Word Sense Disambiguation (NLM WSD) data set [6], consists of only 50 frequently occurring ambiguous terms from the 1998 MEDLINE^® ^baseline. Each ambiguous term in the data set contains 100 instances where each instance was manually assigned a sense from the 1999 Unified Medical Language System (UMLS^®^) Metathesaurus^® ^or *None of the above *if no candidate was acceptable.

The reason manually curated collections contain a limited number of entities is due to the amount of time and resources required to build them. To help alleviate this disadvantage, a number of methods have been developed to produce data sets that do not require manual curation and therefore can be more easily updated.

One method, discussed by Manning and Schütze [7], is called pseudo(conflated)-words. In this method, annotated data is collected by selecting two non-ambiguous terms and turning them into a single ambiguous term. Pedersen [8] evaluated his unsupervised word sense discrimination method on a data set created using this technique. The data set consisted of 60 pseudo-words created from the biomedical domain. As noted by the author, the disadvantage of this technique is that the distinction between the context of the terms is coarse, making it impossible to evaluate a method's performance on finer grained distinctions. Another method uses a multi-lingual corpus where an ambiguous term in one language is not ambiguous in another [9]. In this method, the sense of the unambiguous term in the one language is assigned to the ambiguous term in the second language. This method could be applied to the biomedical domain using a corpus such as Wikipedia. The disadvantage of this method though is that senses in corpora such as Wikipedia do not currently align with concepts from the UMLS.

Liu, Teller and Friedman [10-12] automatically created an abbreviation disambiguation data set consisting of 35 three-letter abbreviations using synonym information from the UMLS. The data set was later re-created for 21 of the 35 three-letter abbreviations by Stevenson, et al. [13] using the method proposed by Schwartz, et al. [14] in which the expansion is identified in the data set and replaced with the abbreviation. The disadvantage of this method is that it will not work for creating a data set that also contains ambiguous terms, and only can be used to create an abbreviation data set where the expansions are explicitly identified within the text.

In this paper, we propose a method that automatically extracts instances of ambiguous terms from MEDLINE without manual curation which also uses MeSH^® ^indexing of MEDLINE as a resource. We have developed a WSD data set, which we refer to as MSH WSD. The resulting data set contains both biomedical terms and abbreviations and is automatically created using the UMLS Metathesaurus and the manual MeSH indexing of MEDLINE.

Fan and Friedman [15] previously explored generating a clinically focused WSD dataset based on the MeSH indexing of MEDLINE. They manually evaluated the creation of this dataset showing the potential for using MeSH indexing of Medline as a resource to automatically creating WSD datasets. The method proposed by Fan and Friedman focuses on obtaining clinically oriented terms, where our focus is broader, encompassing both clinical and biomedical terms as well as abbreviations. Due to our goal of creating a broader data set, our method applies different filtering techniques in order to ensure the reliability of the annotations.

In the remainder of this paper, we first describe the UMLS and MEDLINE. Second, we describe our method to generate the MSH WSD corpus and compare it to the NLM WSD corpus. Third, we use the MSH WSD data set to evaluate four knowledge-based disambiguation methods and analyze the results.

### Unified Medical Language System

The UMLS [16,17] is a knowledge representation framework designed to support biomedical research. It includes over 100 controlled medical terminologies [18] such as the Systematized Nomenclature of Medicine-Clinical Terms (SNOMED-CT) and Medical Subject Headings (MeSH). The three major components of UMLS are the Metathesaurus, Semantic Network and SPECIALIST Lexicon.

• The Metathesaurus is a multi-lingual lexical database that semi-automatically integrates information about biomedical and health-related concepts from biomedical and clinical sources [19] under a common representation. The Metathesaurus creates concepts from the various sources and assigns each concept a Concept Unique Identifier (CUI). A CUI may refer to multiple terms from the individual terminologies. These concepts are labeled with Atomic Unique Identifiers (AUIs). For example, the AUI Cold Temperature [A15588749] from MeSH and the AUI Low Temperature [A3292554] from SNOMED-CT are mapped to the CUI Cold Temperature [C0009264]. As of UMLS version 2009AB the Metathesaurus contains around 1.5 million concepts. Ambiguity arises in the Metathesaurus when a term maps to more than one CUI. For example, the term *cold *maps to the CUIs Cold Temperature [C0009264], the Common Cold [C0009443], Cold Sensation [C0234192], Chronic Obstructive Lung Disease [C0024117], or Colds homeopathic medication [C1949981] which meaning is correct depends on the context in which the term is used.

• The Semantic Network provides a categorization of Metathesaurus concepts into semantic types and relationships between semantic types. A semantic type is a cluster of concepts that are meaningfully related in some way. For example, the semantic type of *Cold Temperature *is *Natural Phenomenon or Process*, whereas *Temperature *is assigned the semantic type *Quantitative Concept*. A concept may be assigned more than one semantic type. For instance, the CUI *C0023175 (lead) *is a *Hazardous or Poisonous Substance *and an *Element, Ion, or Isotope*.

• The SPECIALIST Lexicon contains English biomedical terms and general English terms that are used in the biomedical and health-related domains. The SPECIALIST Lexicon is supplemented with Natural Language Processing (NLP) tools such as the SPECIALIST minimal commitment parser and lexical variation generator (LVG).

### Concept Unique Identifiers

CUIs in the Metathesaurus denote possible senses that a term may have in the Metathesaurus. A CUI is expressed by specific attributes that define it such as its:

• preferred term

• associated terms (synonyms)

• concept definition

• related concepts

For example, the CUI C0009264 has the preferred term *Cold Temperature*. The definition of *Cold Temperature [C0009264] *is:

Having less heat energy than the object against which it is compared; the absence of heat

Some of the terms associated with *Cold Temperature [C0009264] *are:

• Cold Temperature

• Low Temperature

• Cold Thermal Agent

• Cold

Metathesaurus terms that are commonly used to describe the concept include the preferred term in its list. This is where cases of ambiguity arise: the term *cold *is associated with more than one CUI in the Metathesaurus: Cold Temperature [C0009264], the Common Cold [C0009443], Cold Sensation [C0234192], Chronic Obstructive Airway Disease [C0024117], or Colds homeopathic medication [C1949981].

There are two different types of relations that can exist between concepts, subsumption relations (is-a) such as parent/child, and other relations such as siblings. For example, the parent of Cold Temperature [C0009264] is Temperature [C0039476] and one of its siblings is Hot Temperature [C2350229].

The terms, depending on the availability, are represented in several languages, although only English terms are used in this work. Due to the National Library of Medicine's (NLM) focus on source transparency, virtually all the information related to a concept can be traced back to the resource from where it was collected.

### MEDLINE

MEDLINE is an abbreviation for *Medical Literature Analysis and Retrieval System Online*. It is a bibliographic database containing over 18 million citations to journal articles in the biomedical domain which is maintained by NLM. Currently, the citations come from approximately 5,400 journals in 39 different languages starting from 1947. The majority of the publications are scholarly journals but a small number of newspapers, magazines, and newsletters have been included.

MEDLINE is manually indexed with Medical Subject Headings (MeSH). MeSH is NLM's controlled vocabulary thesaurus and consists of sets of term descriptors called *MeSH headings*. These headings are organized in a hierarchical structure where the most general level of the hierarchy contains broad headings such as *Anatomy *or *Mental Disorders*, and the more specific level contains narrow headings such as *Ankle *or *Conduct Disorder*. Currently, MeSH contains 25,588 MeSH headings and over 172,000 entry terms to assist the indexers in determining the appropriate MeSH headings to assign to a MEDLINE citation. MeSH is one of the sources that is included in the UMLS Metathesaurus. The headings in MeSH are not ambiguous because they are created specifically to provide indexing terms for MEDLINE, but when MeSH is incorporated into the Metathesaurus with other sources, ambiguity is introduced. For example, the MeSH headings *Drinking *(e.g. drinking water) associated with the CUI *C0684271 *and *Alcohol drinking *associated with the CUI *C0001948 *are unambiguous, but when incorporated into the UMLS, both concepts are given the associated term *drinking*, making the term *drinking *ambiguous.

#### Analysis of Ambiguity in MEDLINE

Metathesaurus concepts are associated with one or more of 133 semantic types defined in the UMLS Semantic Network. These semantic types are grouped into 15 semantic groups, which define a higher level categorization. A full list of semantic types and the semantic groups can be found at [20].

Table 1 shows the distribution of ambiguous terms in each of the semantic groups. For example, the semantic group Geographic Areas (GEOG) contains the term *Java *which could refer to the island but it also could refer to the programming language. Similarly, the semantic group Genes & Molecular Sequences (GENE) contains the term *adenomatous polyposis coli *which could refer to the gene or the disease. The results in this table show that the semantic group Concepts & Ideas (CONC) has a large proportion of ambiguous terms.

**Table 1:** Distribution of ambiguous terms per semantic groups

Group	Description	Distinct	Ambiguous	% ambiguous	MeSH
ACTI	Activities & Behaviors	7652	236	3.08	12
ANAT	Anatomy	183049	1328	0.73	182
CHEM	Chemicals & Drugs	1043202	15015	1.44	503
CONC	Concepts & Ideas	49701	3482	7.01	197
DEVI	Devices	40454	548	1.35	25
DISO	Disorders	230779	4574	1.98	354
GENE	Genes & Molecular Sequences	183096	15724	8.59	302
GEOG	Geographic Areas	1835	445	24.25	190
LIVB	Living Beings	433254	2475	0.57	141
OBJC	Objects	11658	577	4.95	36
OCCU	Occupations	3559	240	6.74	16
ORGA	Organizations	3939	175	4.44	18
PHEN	Phenomena	9903	240	2.42	18
PHYS	Physiology	307357	4437	1.44	80
PROC	Procedures	327686	1760	0.54	155

This table shows the term ambiguity per semantic group. The row "Distinct" denotes the number of unique terms which belong to any concept assigned to the given semantic group. The row "Ambiguous" denotes the number of unique terms which are assigned to more than one concept of the given semantic group.

Table 2 shows the intersection between the terms of the different semantic groups. The intersection of the groups Chemicals & Drugs (CHEM) and GENE has the largest number of ambiguous terms, which is expected because terms for proteins under CHEM and genes under GENE often share similar terminology. The table also shows that the group *CONC *seems to have larger intersections with the other groups.

**Table 2:** Intra-semantic group ambiguity

	ACTI	ANAT	CHEM	CONC	DEVI	DISO	GENE	GEOG	LIVB	OBJC	OCCU	ORGA	PHEN	PHYS	PROC
ACTI	7652	3	13	158	3	75	17	3	12	10	2	4	14	29	48
ANAT		183049	307	311	37	182	181	38	56	24	6	8	7	48	121
CHEM			1043202	425	235	384	9674	124	808	224	9	25	45	827	518
CONC				49701	144	374	366	214	397	697	122	75	81	599	589
DEVI					40454	53	36	3	12	54	0	1	2	17	67
DISO						230779	2077	89	148	38	14	24	55	372	237
GENE							183096	186	111	56	12	31	24	461	308
GEOG								1835	45	9	0	8	8	36	51
LIVB									433254	140	70	32	6	40	82
OBJC										11658	8	931	5	19	33
OCCU											3559	11	6	14	47
ORGA												3939	1	17	28
PHEN													9903	82	34
PHYS														307357	316
PROC															327686

## Methods

### Data Creation Method

In this section, we describe the method we used to create the MSH WSD data set. This data set consists of instances of MEDLINE abstracts in which each instance contains an ambiguous term that has been assigned a CUI from the UMLS Metathesaurus. This data set was generated without manual annotation, but instead uses existing annotation from MEDLINE citations to annotate the instances of the ambiguous terms with their appropriate CUI (sense).

The development of the MSH WSD data set consists of three steps: ambiguous term identification, citation (instance) retrieval, and quality assurance. We discuss each step in detail.

#### Ambiguous Term Identification

In this step, we identify potentially ambiguous terms from the Metathesaurus which can be assigned a concept from MeSH. To do this, we first extracted terms from the MRCONSO [21] table in the Metathesaurus which have more than one concept associated with them. In the MRCONSO table, each row is an occurrence of a unique term or concept name from each of the source vocabularies. We identified the ambiguous terms from the Metathesaurus by querying for terms in English (field LAT in MRCONSO with value *ENG*) with more than one CUI assigned to them. Table 3 shows an example of the term *lens *and the CUIs assigned to it.

**Table 3:** Example of CUIs assigned to the string lens

CUI	STR	Example of UMLS preferred term
C0023308	lens	Lens Diseases
C0023317	Lens	Lens, Crystalline
C0023318	Lens	Lenses
C0996842	Lens	Genus Lens

Second, we checked which of the CUIs assigned to the terms come from the MeSH vocabulary and disregarded all those that did not. This was done by filtering out those CUIs in MRCONSO in which the SAB field was not equal to the value *MSH*. Examples of words which are ambiguous in the UMLS but were not selected are *frequent, effect *and *study*.

Third, we checked which of the CUIs assigned to the terms are MeSH main headings (MH) and disregarded all those which were not. This was done by filtering out those CUIs in MRCONSO in which the TTY field was not equal to the value *MH*. Table 4 shows an example of the MeSH headings assigned to the CUIs for *lens*. Most of the terms which are not MeSH main headings are substances or qualifiers like *death rate*.

**Table 4:** Example of CUIs assigned to the term lens

CUI	STR	SAB	TTY
C0023308	Lens Diseases	MSH	MH
C0023317	Lens, Crystalline	MSH	MH
C0023318	Lenses	MSH	MH

The information is taken from MRCONSO table fields and shows CUIs linked to the term *lens *and the MeSH term linked to it. CUI is the concept identifier in the Metathesaurus, STR is the MeSH Heading linked to the concept, SAB indicates the source of the string which is MeSH in this case, TTY indicates that the strings in the table are MeSH Headings.

This process of ambiguous term identification resulted in 1,031 ambiguous terms where each term has at least two possible concepts linked to MeSH headings.

#### Citation Retrieval

We retrieve MEDLINE citations from PubMed^® ^[22] containing our ambiguous terms given automatically generated queries using the ambiguous terms and the MeSH headings associated with the term. These citations act as instances of the ambiguous terms. PubMed uses a boolean query language and allows the search to be constrained by specifying specific fields. We use the following specification to constrain our search:

• Constraint: Require the ambiguous term to appear in the title or the abstract of the citations.

- Implementation: We do this by using the tag *TIAB*.

• Constraint: Require both the MeSH heading and the ambiguous term to be associated with the citation.

- Implementation: We do this by combining the MeSH header with the ambiguous term using the AND operator and also use the tag *MH *to indicate that the search is conducted on the MeSH headings.

• Constraint: Ensure descendants of the MeSH headings are not returned.

- Implementation: MeSH is organized in a taxonomy and PubMed searches with the MeSH heading and any descendants. To avoid descendants of the MeSH heading being returned, we constrain our search using the *MH:noexp *tag, which increases the specificity of our query.

• Constraint: Ensure only one of the MeSH headings associated with the ambiguous term is assigned to the citation.

- Implementation: To do this, we combine the possible MeSH headings of the ambiguous term using the NOT boolean operator and the *MH:noexp *tag.

**Figure 1:**
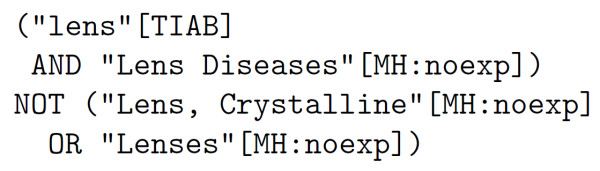
**Example query for one of the senses of term lens**. PubMed query used to retrieve citations which contain the term *lens *when it is related to *lens diseases*. The retrieved citations should have been indexed with the MeSH Heading *lens diseases *and should not be indexed with *Lens, Crystalline *or *Lenses*.

An example query for the ambiguous word, *lens*, can be found in Figure 1.

#### Quality assurance

Some queries in the citation retrieval step returned very few citations, and some of the citations returned were not representative of the senses in the Metathesaurus. We have made assumptions similar to Fan and Friedman [15]: each ambiguous term will have only one sense per MEDLINE citation and the sense is assigned to the overlapping MeSH heading. These assumptions are prone to incorrect annotations, therefore we also created three filters to verify that enough examples were returned to ensure that the term is clearly used with a distinctive sense.

**Figure 2:**
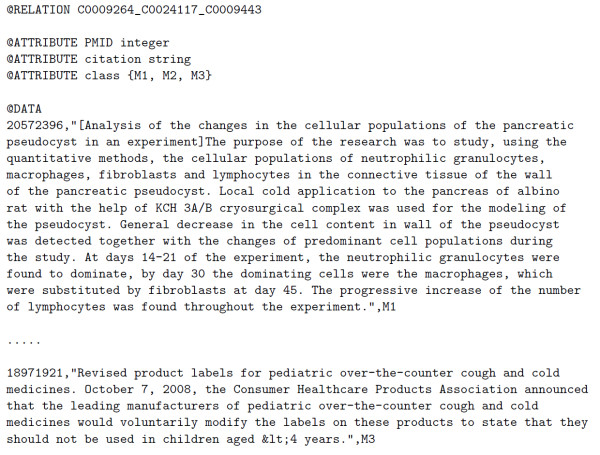
**WSD example for the term cold in ARFF format**. The *@RELATION *line contains the list of concepts from the Metathesaurus. Each data line has the PMID of the citation, the text where the ambiguous term appears and the sense number.

• **Step 1**

- Filter: Avoid cases where, for a given sense of a term, there is a low number of (or no) occurrences linked to a MeSH heading in MEDLINE.

- Implementation: We select only cases where at least 10 citations have been retrieved for each one of the senses. The terms which do not comply with this criterion have been filtered out.

- Result: A total of 592 citations passed this filter out of the original 1,031.

• **Step 2**

- Filter: Avoid senses that are not distinct enough, which might mean that, for instance, both concepts should be merged in the UMLS or that hypernym terms appear in the hyponyms. An example is the term *sodium*, which may refer to the element, or a high/low sodium diet, but in all the cases in the retrieved citations the term *sodium *makes reference to the element. The goal of this task it to limit the amount of manual intervention; therefore, we conducted an assessment over the remaining terms using statistical learning methods.

- Implementation: We created a learning model using the Support Vector Machine from the WEKA datamining package [23] and the text of the citations retrieved for each instance as the context for disambiguation. We average the recall results using 10-fold cross validation and discard the ambiguous terms for which the recall was lower than 0.8 for any sense. We preferred recall to F-measure to avoid cases where you have high precision and low recall in one sense and high recall but low precision in another, which would mean that most of the sense annotations are wrongly assigned to one of the senses. An example is available in the Weka file format (ARFF) [24] and can be seen in Figure 2.

- Result: A total of 211 out of the 592 citations passed this filter.

• **Step 3**

- Filter: Avoid noisy cases which did not contribute to the goal of the data set.

- Implementation: We remove terms consisting of only one letter.

- Result: A total of 203 out of the 211 citations passed this filter

### MSH WSD data set

The MSH WSD data set consists of 203 ambiguous entities in which 106 out of the 203 ambiguous entities are abbreviations (indicated by an A in the table), 88 are strictly terms (T) and 9 are a mixture of both (B). See additional file 1: Accuracy per ambiguous word. This additional file, among other results, shows the resulting MSH WSD data set. For example, in our abbreviation subset, the term *CAD *refers to either a *Coronary Artery Disease *or *Computer Assisted Diagnosis*, and the term *pI *refers to either an *S-Phase Fraction *and *Isoelectric Point*. An example of a mixture of abbreviation and terms is the term *Eel *which refers to either the animal or *Electron Energy-Loss Spectroscopy*. In this paper, we do not make a distinction between abbreviations and acronyms.

### Comparison to the NLM WSD set

In this section, we compare the characteristics of the MSH WSD data set to that of the NLM WSD data set. We would like to highlight that the NLM WSD set was generated based on the tagset from the 1999 version of the Metathesaurus which has significantly changed over time due to the addition, splitting and converging of concepts with each new addition of the Metathesaurus that is released.

The NLM WSD dataset also contains the annotation *None of the above*, if the sense was not in the Metathesaurus. The senses of the instances annotated as *None of the above *may currently be covered by subsequent versions of the Metathesaurus, but not at the time of its creation.

The MSH WSD data set does not have a *None of the above *category to denote cases where the sense of the ambiguous term in the text cannot be mapped to the Metathesaurus. This is due to the fact that the NLM WSD data set was manually curated, and therefore, a human had the option of stating that none of the senses in the UMLS apply to the instance containing the target word. The MSH WSD data set is automatically generated and does not have the manual intervention. In addition, the filters applied to the MSH WSD set will remove terms with a small number of occurrences. As explained above, we favored high precision to high coverage removing sporadic mentions which might contribute to possible misannotations in the data set.

As stated above, in the MSH WSD data set, there exist 106 terms that are actually abbreviations and 9 that are a mixture of both. In the NLM WSD data set only the ambiguous term *cold *has an acronym for a possible sense; one of its senses is *Chronic Obstructive Lung Disease (COLD) *where the other the possible senses are Cold Temperature, Common Cold, Cold Therapy, and Cold Sensation.

The terms, *cold, ganglion *and *radiation*, exist in both the NLM WSD and MSH WSD data sets. Although in the MSH WSD data set, the term *cold *has only three senses: *Cold Temperature, CommonCold *and *COLD*, related to *Chronic Obstructive Lung Disease*; the senses related to *Cold Therapy, Cold Sensation *and *Cold as a Pharmacologic Substance *are not included because they do not exist in MeSH. The senses for the terms *ganglion *and *radiation *are the same in both data sets.

The semantic type coverage in the MSH WSD data set is broader than that in the NLM WSD data set. The possible senses of the target words in the MSH WSD data set encompass 81 out of 133 semantic types in the UMLS, whereas the NLM WSD data set only covers 46 semantic types. See additional file 2: Semantic Type frequency in the MSH WSD set and Metathesaurus concept count. It shows the full list of semantic types which appear in the MSH WSD data set with the number of concepts in the Metathesaurus linked to that semantic type.

Table 5 shows the top 10 semantic types in each set. The table shows that the NLM WSD data set has a large set of possible senses which belong to the Semantic Group *Concept and Ideas *(*CONC*) while the MSH data set contains more biomedically grounded terms.

**Table 5:** Top semantic types by frequency in the NLM WSD and our data set

NLM WSD			MSH WSD		
**Type**	**Description**	**Freq**	**Type**	**Description**	**Freq**.

T061	Therapeutic or Preventive Procedure	9	T047	Disease or Syndrome	73
T040	Organism Function	7	T116	Amino Acid, Peptide, or Protein	50
T032	Organism Attribute	7	T121	Pharmacologic Sub-stance	44
T098	Population Group	6	T123	Biologically Active Substance	32
T070	Natural Phenomenon or Process	6	T023	Body Part, Organ, or Organ Component	29
T041	Mental Process	6	T109	Organic Chemical	26
T081	Quantitative Concept	6	T083	Geographic Area	24
T080	Qualitative Concept	6	T129	Immunologic Factor	17
T059	Laboratory Procedure	5	T191	Neoplastic Process	15
T170	Intellectual Product	5	T114	Nucleic Acid, Nucleoside, or Nucleotide	11

For each set, the semantic type, description and frequency in the set are shown.

In the MSH WSD data set, the instances for each possible concept in the data set are balanced. This means that for each ambiguous term, we have approximately the same number of instances per possible sense. Balancing allows a better evaluation of algorithms which may be influenced by a skewed distribution of senses. Specifically, supervised machine learning algorithms which learn a model based on the instances of the dataset; an unbalanced set has the potential to create a model influenced by the fact the majority of the senses in the training data are annotated with a single sense. On the other hand, usually senses are skewed, but estimation of the prior distribution of senses of an ambiguous word in a corpus is difficult to approximate. We have provided the frequency of the MeSH term in MEDLINE which could be used as an indicator of its sense proportion. See additional file 3: Sense frequency and MeSH Heading.

In the NLM WSD data set, the distribution of terms is obtained randomly from the 1998 MEDLINE citations and is not balanced. For example, the term *transport* has two possible senses *Biological Transport *and *Patient Transport*. The NLM WSD data set contains 93 out of 100 instances of *Biological Transport*, one instance of *Patient Transport *and six instances which were annotated as *None of the above *indicating that neither sense applies.

We queried MEDLINE using PubMed on the 23rd of July 2010, the precise date the corpus was generated. For each term, we retrieved the total number of occurrences, see additional file 3: Sense frequency and MeSH Heading. As shown in Table 6 there are terms covering a large set of frequencies in MEDLINE. We found a large gap from the first term *cell *with 1,903,168 and the term *sodium *with 239,623. The term from the MSH WSD data set with least number of occurrences in MEDLINE is *CPDD *with 59. In the NLM WSD set, the top 24 of the terms ranked by MEDLINE frequency are between these two terms. The term with the lowest frequency is *mole *with 12,947 occurrences.

**Table 6:** NLM WSD term frequency

Term	Frequency
single	830940
growth	780721
evaluation	626911
surgery	602878
reduction	547831
inhibition	525793
pressure	492250
support	470918
weight	470011
frequency	460948
sensitivity	410728
failure	375471
culture	365909
resistance	355190
degree	338131
determination	307813
energy	281706
lead	280893
glucose	265023
scale	263109
strains	255978
sex	255545
condition	251454
uid	249806
variation	228733
secretion	222020
transport	219625
man	205108
radiation	199449
blood pressure	181752
transient	175823
white	174704
depression	165689
repair	158033
pathology	146981
fat	133861
extraction	121110
ultrasound	115408
discharge	89344
implantation	87057
nutrition	80029
adjustment	71935
japanese	67796
cold	67218
fit	55692
ganglion	42474
immunosuppression	32835
mosaic	19621
mole	12947

Frequencies of the terms is MEDLINE as of 23rd July 2010.

To summarize, the MSH WSD data set covers a larger set of the UMLS Semantic Types compared to the NLM WSD data set. In addition, the MSH WSD data set is simply larger and provides a broader range of biomedical terms and acronyms. The frequency of those terms varies and does not consist of only the very frequent ones. Furthermore, the MSH WSD data set has been generated automatically reusing already existing annotations and can be regenerated every time a new UMLS version becomes available.

### Description of Existing WSD Methods

In this section, we compare different WSD methods on our MSH WSD data set. These comparisons allow us to validate the usability of the data set. We consider a statistical learning method and five previously reported knowledge-based methods which have been used to disambiguate terms in the original NLM WSD data set, their performance is shown in table 7:

**Table 7:** Overall accuracy on the data set

Data set	NB	AEC	JDI	MRD	2-MRD
Abbreviation Set	0.9716	0.9090		0.8759	0.8501
Abbreviation Subset	**0.9760**	0.9218	0.6725	0.8838	0.8725

Term Set	0.8980	0.7462		0.7148	0.6773
Term Subset	**0.8991**	0.7448	0.6209	0.7132	0.6609

Term/Abbreviation Set	**0.9384**	0.8879		0.8801	0.9356
Term/Abbreviation Subset	0.9360	0.9026	0.6899	0.8715	0.9350

Overall MSH WSD Set	0.9386	0.8383		0.8070	0.7799
Overall MSH WSD Subset	**0.9413**	0.8448	0.6551	0.8118	0.7837

NLM WSD	0.8830	0.6836		0.6389	0.5500
NLM WSD Subset	0.9063	0.6932	0.7475	0.6526	0.5800

NB stands for Naïve Bayes, AEC stands for Automatic Extracted Corpus, MRD stands for Machine Readable dictionary, 2-MRD stands for 2nd Order Co-occurrence MRD, and JDI stands for Journal Descriptor Indexing. The term set stands for all the ambiguous words in the category while subset indicates that only the words that the JDI method can use are considered. Results on the NLM WSD set have been included.

• Supervised Naïve Bayes

• Automatic Extracted Corpus (AEC) [25]

• Journal Descriptor Indexing (JDI) [26]

• Machine Readable Dictionary (MRD) [25,27]

• 2nd Order Co-occurrence MRD (2-MRD) [28]

Our research focus in WSD methods is on knowledge-based methods. We include the supervised learning method to provide a top end baseline, this method is based on statistical learning and a similar method used in Step 2 of the quality assurance step. The advantage of supervised learning methods is that they typically assign senses to ambiguous terms with a high degree of accuracy. The disadvantage is that they require training data for each term that needs to be disambiguated, whereas knowledge-based methods, although historically obtain a lower accuracy, do not require training data.

#### Supervised Naïve Bayes (NB) Method

In the supervised Naïve Bayes Method, we use the words occurring in the text of the citation where the ambiguous term appears as features in a supervised Naïve Bayes algorithm from the WEKA datamining package [23]. We report the Naïve Bayes result using WEKA's 10-fold cross-validation.

#### The Automatic Extracted Corpus (AEC) Method

The Automatic Extracted Corpus (AEC) Method attempts to alleviate the problem of requiring manually annotated training data for supervised learning algorithms. In this method, training data is automatically generated and is used to train a machine learning algorithm to disambiguate ambiguous terms.

The training data is automatically generated using documents from MEDLINE [29]. To create the training data, we automatically generate queries using English *monosemous relatives *[30] of the possible senses which, potentially, have an unambiguous use in MEDLINE. The list of candidate relatives include synonyms and terms from related concepts. Documents retrieved using PubMed are assigned to the concept which was used to generate the query. If no documents are returned for a given query, quotes are replaced by parentheses to broaden the search and allow finding the terms in any position in the title or abstract. The automatically generated training data is then used to train a Naïve Bayes classifier using the words surrounding the ambiguous term in the citation as features. The model is then used to disambiguate the ambiguous term in the MSH WSD data set.

#### Journal Descriptor Indexing (JDI) Method

The JDI Method, introduced by Humphrey, et al. [26], automatically assigns a concept to an ambiguous term by first identifying its semantic type with the assumption that each possible concept has a distinct semantic type. In this method, a semantic type vector is created for the semantic type of each of the possible concepts using one-word terms in the UMLS. A vector representing the ambiguous term is created using the words that exist in the same citation as the ambiguous term. The angle between this vector and each of the semantic type vectors is calculated using the cosine measure. The concept whose semantic type vector is closest to the vector representing the ambiguous term is assigned to the term. As this method relies on the semantic type(s) assigned to a concept, if two or more of the possible senses are assigned the same semantic type, this algorithm cannot disambiguate the ambiguous term. The JDI experiments in this paper were conducted using the JDI implementation of this method and is available as part of the SPECIALIST Text Categorization tools [31].

#### The Machine Readable Dictionary (MRD) Method

The MRD method creates a context vector by extracting the content words surrounding the ambiguous word, and compares it to a profile vector built for each of the UMLS concepts linked to the ambiguous term being disambiguated. Vectors of concept profiles linked to an ambiguous word and word contexts are compared using cosine similarity. The concept with the highest cosine similarity is selected. This method has been previously used by McInnes [27] in the biomedical domain with the NLM WSD data set.

A concept profile vector has as dimensions the tokens obtained from the concept definition, or definitions, if available, of synonyms and of related concepts (excluding siblings). Stop words are discarded, and Porter stemming is used to normalize the tokens. In addition, the token frequency is normalized based on the inverted *concept *frequency so that tokens which are repeated many times within the UMLS will have less relevance.

In order to perform disambiguation, the context of the ambiguous term is turned, as well, into a vector representation. The context vector for an ambiguous term includes the term frequency. The stop words are also removed, and the Porter Stemmer is applied. The word order, as in the concept profile, is lost in the conversion.

#### 2nd Order Co-occurrence Machine Readable Dictionary (2-MRD) Method

The 2-MRD Method, introduced by McInnes [28], uses second-order co-occurrence vectors to represent the ambiguous term and each of its possible concepts. This is similar to the MRD method above except that the vectors used to represent the ambiguous terms and concepts are second-order co-occurrence vectors rather than the first-order co-occurrence vectors.

In this method, the ambiguous term is created by first creating a co-occurrence matrix in which rows represent the words surrounding the ambiguous term, and the columns represent words that co-occur in a corpus with those words. Each cell in this matrix contains the frequency in which the word found in the row occurs with the word in the column. Second, each of the words surrounding the target word is replaced by its corresponding vector as given in the co-occurrence matrix, and the centroid (averaged vector) of these vectors is the second-order co-occurrence vector used to represent the meaning of the target word.

The vectors for each possible concept (concept profile vectors) are created in a similar fashion by using the words in the concept's definition as well as the definitions of its related concepts. The cosine is calculated between the vector representing the target word and each of the vectors representing the possible concepts. The possible sense whose vector is the closest is mapped to the term. The 2-MRD experiments in this paper were conducted using CuiTools v0.15, which is a freely available open source package [32].

## Results and Discussion

Table 7 shows the overall results of the MSH WSD data set. The data set is broken into three sections: Abbreviation Set, Term Set and the Term/Abbreviation Set. The Abbreviation Set contains 106 ambiguous acronyms identified as A in the additional file 1: Accuracy per ambiguous word. The Term set contains 88 ambiguous terms, identified as T, and the Term/Abbreviation Set contains 9 ambiguous term/abbreviations, identified as AT.

Since the JDI method is only able to disambiguate ambiguous terms or abbreviations whose possible senses do not share the same semantic type, we created additional subsets for comparison. There exist 44 ambiguous terms in which this method is not able to distinguish between the possible senses. The results for the individual terms and abbreviations can be seen in the additional file 2: Accuracy per ambiguous word.

Considering these three categories, the T term set is more difficult to disambiguate for all of the methods presented here. This indicates that the contextual difference between ambiguous terms is finer grained than the contextual differences between abbreviations.

We compared the statistical significance for each pair of methods using randomization tests [33]. We found that all the differences are statistically significant (*p *<= 0.005).

Table 7 shows, as well, the disambiguation performance achieved by the presented methods evaluated on the NLM WSD corpus. Generally, the performance of the methods is higher in the MSH WSD set. We believe that there are several reasons for this. First, proportionally, there are less instances belonging to the *CONC *(Concept & Ideas) semantic group. Only 2% of the possible senses of the ambiguous terms in the MSH WSD dataset belong to *CONC *versus 21% in the NLM WSD dataset. Concepts from the *CONC *group are, usually, more difficult to disambiguate due to their vagueness. The MSH WSD set provides a larger set of examples from a wider range of semantic groups. The possible senses in the MSH WSD dataset are distributed over 15 different semantic groups versus 12 for the possible senses in the NLM WSD datset. Table 8 shows the distribution of semantic groups for each of the datasets.

**Table 8:** Distribution of semantic groups in the MSH WSD and NLM WSD datasets

Semantic Group(s)	NLM WSD		MSH WSD	
	**frequency**	**percentage**	**frequency**	**percentage**

Activities & Behaviors	7	0.0619	5	0.0121
Anatomy	4	0.0354	44	0.1063
Chemicals & Drugs	3	0.0265	118	0.2850
Concepts & Ideas	24	0.2124	10	0.0242
Devices	1	0.0088	6	0.0145
Disorders	13	0.1150	100	0.2415
Living Beings	7	0.0619	39	0.0942
Objects	2	0.0177	3	0.0072
Occupations	3	0.0265	3	0.0072
Phenomena	9	0.0796	4	0.0097
Physiology	20	0.1770	15	0.0362
Procedures	20	0.1770	28	0.0676
Genes & Molecular Sequences	0	0	8	0.0193
Geographic Areas	0	0	23	0.0556
Organizations	0	0	5	0.0121
Chemicals & Drugs/Objects	0	0	2	0.0048
Objects/Organizations	0	0	1	0.0024

In addition, the MSH WSD test set is balanced, this means that there is the same number of instances for each sense, in contrast to the NLM WSD set where in some cases there were only one example instances for some of the senses, so methods were not evaluated on all of the possible senses. In addition, studies for the NLM WSD set are usually done on the 1999 version of the UMLS while the study with the MSH WSD set is based on the 2010AB version of the UMLS which might provide additional information to the knowledge-based disambiguation algorithms.

Generally, all of the methods obtain a higher accuracy in disambiguating ambiguous terms from the Abbreviations set than the Term set. Since the long form of the abbreviation might be present in the abstract, this itself could provide enough context for the algorithms to disambiguate between them. All of the methods found the ambiguous term *SS *from the Abbreviation Set easy to disambiguate but found *HIV *from the same set much more difficult.

As expected the supervised Naïve Bayes method obtained a higher overall disambiguation accuracy than the knowledge-based methods. The AEC method obtains the second highest with the exception of the Term/Abbreviation subset where 2-MRD obtained the second highest accuracy. Analysis of these results shows that abbreviations obtained the highest accuracy overall.

In the AEC method, the ambiguous terms and abbreviations that obtained the lowest accuracy are *Erythrocytes, RBC, DE, Cortex *and *Pneumocystis*. The senses associated with *erythrocytes *and *RBC *both refer to the substance and to the count of the substance as a result of an analysis. The distinction between the senses is very fine grained, and therefore the queries generated did not retrieve relevant training data. The possible senses for DE are *Delaware *and *Germany*. The queries generated with the names of the country did not retrieved relevant documents. *DE *and the name of the country/region do not have to appear together. In the case of *cortex*, noisy terms related to *adrenal cortex *have retrieved documents related to *cerebral cortex*, e.g. *chemically induced*. The term *pneumocystis *has two very close senses, either a fungus or a pneumonia caused by this fungus. The Metathesaurus terms for each one of the possible senses are not discriminating and have retrieved citations for both senses.

The JDI method obtains the lowest disambiguation accuracy. This is surprising compared to previously published work [25]. Many of the accuracies are close to 0.50 indicating that the method has a preference for one of the senses; see additional file 1: Accuracy per ambiguous word. There are several reasons for this behavior which have been described in [25] when evaluated on the NLM WSD data set. These reasons are mainly related to the granularity of JDs used to index the semantic types, and the context of the ambiguous word. The NLM WSD contains a smaller number of semantic type combinations which seem to perform reasonably well; but in our data set, the combination is larger and includes semantic types with a smaller number of sample terms in the Metathesaurus. The ambiguous terms/abbreviations with the lowest disambiguation accuracy are *Fe*, lens *and TAT *with an accuracy of less than 0.40. See additional file 3: Sense frequency and MeSH Heading. This additional file shows the ambiguous terms/abbreviations with their possible senses and the number of citations that the MeSH heading has been assigned to a citation in MEDLINE. Note that this is different than the majority sense in the MSH WSD data set itself. The senses of *Fe *refer either to the chemical entity (*M1*) or as part of a diet (*M2*). The semantic types of these senses are related to chemical entities which are not distinct enough for the JDI method to disambiguate accurately. The term *lens *has three possible senses. The MeSH heading referring to the medical device (*M1*) is the majority sense in the MSH WSD data set. This is the sense most often assigned to an instance by the JDI method. Interestingly though, the MeSH heading referring to *Part of the Eye *(*M3*) has the largest number of citations associated with it in MEDLINE but is never assigned to an instance by the JDI method. The term *TAT *has three possible senses. Two of the senses are difficult to distinguish between because one refers to the *gene *and the other to its *product*. The third sense refers to *Thematic Apperception Test *but is never assigned to an instance by the method.

The MRD method obtained a higher overall disambiguation accuracy than 2-MRD. These two methods are similar and mainly differ only in the type of vectors that are created. The MRD method uses first-order co-occurrence vectors whereas the 2-MRD method uses second-order co-occurrence vectors. This indicates that the second-order co-occurrence vectors may be introducing too much noise for the method to distinguish between some of the more finely grained ambiguous terms. The performance of the MRD method shows similar behavior as previously seen in [25], and the performance of 2-MRD shows similar behavior as seen in [28]. Each of the methods rely on the matching between the context of the ambiguous word and context of the concept profiles for each of the possible senses. A mismatch between context in which the terms are used and the concept profiles might cause the method to erroneously assign a possible sense. The terms with the highest disambiguation accuracy for both methods are *PAC, BPD*, and *CLS*. These are abbreviations where there is little overlap between the candidate concepts, and the presence of the long form is distinctive enough to select the correct possible sense.

The terms with the lowest disambiguation accuracy for MRD are *phosphorus, lens *and *Fe *with an accuracy under 0.50. The term *lens *has three senses with the sense *Lens*, *Crystalline *most often occurring in MEDLINE. The MRD method assigns instances to either *Lenses *and *Lens*, *Crystalline*. The terms *phosphorus *and *Fe *refer to either the chemical element or its dietary use. The context surrounding the senses for each of these ambiguous words is not distinct enough to accurately disambiguate between them.

The terms with the lowest disambiguation accuracy with 2-MRD are *THYMUS, Pleuropneumonia*, and *Borrelia*. The senses for the term *THYMUS *refer to the extract, the plant and the gland, with the gland being most referred to in MEDLINE. The context between the extract and the plant is too fine grained for the method to distinguish between them. Similarly, the senses for the term *Pleuropneumonia *and *Borrelia *both refer to either the disease itself or the bacteria which cases the disease. Again, the contexts surrounding each of these terms are very similar, and therefore, do not provide enough distinction for the method to distinguish between them.

The JDI, NB and AEC methods exhibit a similar behavior in all the quartiles, whereas the MRD and 2-MRD methods do not. The MRD and 2-MRD methods obtain a higher disambiguation accuracy when the frequency counts are lower indicating that they are possibly sensitive to too much information or noise, and therefore may require stricter filters to remove features that do not provide distinguishing information when creating their vectors.

To further analyze the results, we extracted the frequencies for each term in MEDLINE and split the set into four equal parts. Table 9 shows the results for each one of the frequency quartiles. For most methods, the first quartile of the most frequent terms in MEDLINE obtains a lower disambiguation accuracy compared to the others. We find in this group terms which are difficult to disambiguate, but are very frequent in MEDLINE like *sodium *which might refer to the element or a component in a diet. Another example is *erythrocytes*, which might refer to either *red blood cell *or its measurement. This last example shows similar characteristics to the target word *blood pressure *in the NLM WSD data set [34].

**Table 9:** Accuracy per ambiguous word MEDLINE frequency range

Q	Frequency range	NB	AEC	MRD	2-MRD	JDI
Q1	1,903,168 - 40,499	**0.9499**	0.7708	0.7427	0.7206	0.6505
Q2	40,425 - 11,033	**0.9401**	0.8591	0.8199	0.7812	0.6458
Q3	10,817 - 3,482	**0.9348**	0.8928	0.8490	0.8192	0.6618
Q4	3,427 - 59	**0.9300**	0.8309	0.8160	0.7974	0.6623

NB stands for Naïve Bayes, AEC stands for Automatic Extracted Corpus, MRD stands for Machine Readable dictionary, 2-MRD stands for 2nd Order Co-occurrence MRD, and JDI stands for Journal Descriptor Indexing.

We also grouped the results based on the semantic type and semantic group of the senses for each of the ambiguous terms and abbreviations in the MSH WSD data set. We analyzed those pairs that contained more than two occurrences. For example, the term *crack *has two possible sense: *crack cocaine *and *tooth fractures*, where the first is in the semantic group *Chemicals & Drugs*, and the latter is in the semantic group *Disorders*. See additional file 4: Inter semantic types results which shows the results grouped based on the semantic type of the senses. Also see additional file 5: Inter semantic groups results which shows the results grouped based on their semantic groups. The results for the semantic groups indicate that some groups are easier to classify than others. For example, the groups *Device-Disorders *obtain a 100% accuracy by all of the methods except for the JDI method. This indicates that the contextual information associated with each of the groups is distinct but the semantic types associated with the groups are not. With respect to the JDI method, the results show that it does very well at disambiguating between *Living Beings-Living Beings, Living Beings-Anatomy, Living Beings-Chemicals Drugs *and *Disorders-Organization*. This indicates that the semantic types in these categories are more distinct than those in, for example, *Anatomy-Devices*, and *Chemicals & Drugs-Genes & Molecular Sequences*.

With respect to the MRD and 2-MRD methods, the results show that for a majority of group pairs, the methods perform similarly. The exception to this is *Disorders-Organizations*, where the MRD method obtained a disambiguation accuracy of 98% where 2-MRD obtained an accuracy of only 55%. This indicates that the second-order contextual information in this group is not as distinct as the first-order information and is unable to distinguish between these two group pairs.

Finally, we combined the knowledge-based disambiguation methods using the same methods presented in [25]. When we combine the methods by summing the their scores (or probabilities), the average disambiguation accuracy is 0.8447. When we combine the methods by voting, the average accuracy is 0.8403. AEC is the best performing method with an average accuracy of 0.8383, indicating that combining the scores of the individual method obtains an improvement in the WSD performance. Removing the JDI approach, which had the lowest performance, decreases the average accuracy of the sum scores to 0.8407, but increases the average accuracy to 0.8551 when using the voting method, which constitutes a higher disambiguation accuracy than any other individual method or combination of methods.

## Conclusions

In this paper, we describe our MSH WSD data set. Compared to existing sets, this data set has the largest UMLS Semantic Type coverage and contains a broader range of biomedical terms. Furthermore, the MSH WSD data set has been generated automatically, reusing already existing annotations, and can be regenerated as new UMLS versions become available. We believe that the creation of this data set constitutes a significant step forward in the area of WSD, and will promote the development of new WSD methods.

We also described our technique in automatically creating this data set. The technique is based on existing annotations and may be useful in the development of new data sets. We foresee the use of this method with existing resources to obtain annotated data automatically in the biomedical domain. Recently, OMIM was added to the UMLS which could help in the disambiguation of diseases and gene names. In addition, existing data sets for genes and proteins, such as UniProt and EntrezGene, could be used to disambiguate genes and protein terms.

In the current data set, only concepts in the Metathesaurus were considered. The disadvantage to this is that there are senses of terms not covered by it, as shown by experience with the NLM WSD data set. Further research is required to provide a proper annotation of ambiguous terms which cannot be treated with the technique presented in this paper.

The MSH WSD data set is available for download at [35]. All the examples are available, and the documentation indicates the examples removed by each filter. This will allow researchers to consider different filters on the extracted set from MEDLINE.

## Authors' contributions

AJ designed and carried out the experiments, participated in the development of the methods and drafted the manuscript. BM designed and carried out the experiments, participated in the development of the methods and drafted the manuscript. AA designed the experiments and reviewed the manuscript. AJ, BM and AA read, commented, and approved the final version of the manuscript.

## Supplementary Material

Additional file 1 **Accuracy per ambiguous word**. Medline Freq. is the frequency of the term in MEDLINE up to 23rd July 2010. NB stands for Naïve Bayes, AEC stands for Automatic Extracted Corpus, MRD stands for Machine Readable dictionary, 2-MRD stands for 2nd Order Co-occurrence and JDI stands for Journal Descriptor Indexing. The possible values for type are: A for abbreviations, T for terms and AT for abbreviations/terms. Click here for file

Additional file 2 **Semantic Type frequency in the MSH WSD set and Metathesaurus concept count**. Click here for file

Additional file 3 Sense frequency and MeSH Heading Click here for file

Additional file 4 **Inter semantic types results**. Click here for file

Additional file 5 **Inter semantic groups results**. Click here for file
